# A Suggestion

**Published:** 1907-09

**Authors:** 


### A SUGGESTION.

The new Glyco-Thymoline Eye Bath, which is constructed from a single piece of aluminum, has been found of exceptional service when used as a vessel to heat hypodermic solutions to the proper temperature. This little hint comes from a physician who has frequently found himself wanting just such a device. The Glyco-Thymoline people will be glad to send you one of these cups if you desire it.

